# Association of Insomnia, Depressive Disorders, and Mood Disorders as Risk Factors With Breast Cancer: A Nationwide Population-Based Cohort Study of 232,108 Women in Taiwan

**DOI:** 10.3389/fonc.2021.757626

**Published:** 2021-10-11

**Authors:** Hui-Pu Liu, James Cheng-Chung Wei, Hei-Tung Yip, Ming-Hsin Yeh

**Affiliations:** ^1^Department of General Surgery, Kaohsiung Armed Forces General Hospital, Kaohsiung, Taiwan; ^2^Department of Allergy, Immunology & Rheumatology, Chung Shan Medical University Hospital, Taichung, Taiwan; ^3^Institute of Medicine, College of Medicine, Chung Shan Medical University, Taichung, Taiwan; ^4^Graduate Institute of Integrated Medicine, China Medical University, Taichung, Taiwan; ^5^Management Office for Health Data, Clinical Trial Research Center, China Medical University Hospital, Taichung, Taiwan; ^6^Department of Surgery, Chung Shan Medical University Hospital, Taichung, Taiwan

**Keywords:** breast cancer, depressive disorders, mood disorders, insomnia, sleeping medication, hyperlipidemia

## Abstract

**Background:**

Insomnia, depressive disorders, and to a more general view, mood disorders are raising people’s concerns and causing disability of life. Herein, we try to seek the association of such illnesses with subsequent breast cancer.

**Methods:**

This population-based, retrospective cohort study used data from the Taiwan National Health Insurance Research Database. This study included 232,108 women diagnosed with insomnia, depressive disorders, and mood disorders from January 1, 2000 to December 31, 2013. Physician diagnosed insomnia, depressive disorders, or mood disorders using outpatient and inpatient records before diagnosis of breast cancer. Cox proportional hazards regression analysis is adjusted for women with insomnia, depressive disorders, mood disorders, and other factors like insured amount, urbanization, and comorbidities such as having subsequent breast cancer.

**Results:**

Sleep medication was associated with a significantly increased incidence rate of breast cancer (aHR = 1.23 (95% CI = 1.13, 1.35), *p* < 0.001). Insomnia was associated with significant increased hazard of breast cancer (aHR = 1.16 (95% CI = 1.07, 1.27), *p* < 0.001). Annual insured amount >20,000 (TWD), high urbanization area, and hyperlipidemia were associated with increased hazard of breast cancer (aHR = 1.13 (95% CI = 1.01, 1.27), *p* = 0.04; aHR = 1.41 (95% CI = 1.17, 1.71), *p* < 0.001; aHR = 1.14 995% CI = 1.02, 1.29), *p* = 0.02, respectively). There was a positive correlation between depressive disorders and increased incidence rate of breast cancer but not statistically significant (aHR = 1.11 (95% CI = 0.99, 1.25), *p* = 0.08). Mood disorders were not associated with increased hazard (aHR = 1.11 (95% CI = 0.91, 1.34), *p* = 0.31).

**Conclusion:**

In this study, women with insomnia had increased risk of breast cancer, particularly those in high urbanization or with high insured amounts. Sleep medication (benzodiazepine (BZD) or non-BZD) and hyperlipidemia were independently associated with a higher hazard ratio of breast cancer. Insomnia along with sleep medication did not yield more hazards than each alone. Mood disorders appeared to be not associated with subsequent breast cancer. However, depressive disorders, the subgroups of mood disorders, could possibly increase the incidence rate of breast cancer though not statistically significant.

## Introduction

Breast cancer, the leading cause of cancer death in women, is drawing more and more attention in health issues universally. There are more than 2 million women with newly diagnosed breast cancer every year globally (1). In Taiwan, 13,965 new cases of breast cancer have been reported in 2017, being the most commonly diagnosed cancer in women: it can be estimated that nearly 27% of cancer cases in women is represented by breast cancer. Breast cancer is the most frequently diagnosed cancer and second most common cause of cancer death in the USA (2), and it is the principal cause of death in women aged 40 to 49 years; in this period, women are in transition of perimenopause and menopause.

Women may experience sleep disturbance, depression, and anxiety during perimenopause and menopause transition due to physiologic changes in responsiveness to gonadotropins with wide variation of hormone level (3). In the late perimenopausal transition lasting 1 to 3 years, most women will encounter amenorrhea for longer than 2 months and often suffer from vasomotor symptoms; during this period and early menopause, FSH level continue to rise while estradiol (E2) level is declining. It is not until 2 years after menopause that the hormone levels remain steady (4). These symptoms due to estrogen decline raise our concern for women’s health about mental illness.

Impairment of cognitive function and affective function after diagnosis of breast cancer were reported (5), and alteration of hippocampus like deformation or volume loss were also found after major types of treatments. Some pivotal studies aimed at the survivorship of returning the quality of life to the status before diagnosis or even better (6). Breast cancer experience could have an impact on physical and psychosocial alterations such as increased risk of depression, anxiety, and intrusive thought, especially in younger patients. Disruption of body image-related (hair loss, weight gain, aesthetics, etc.) distress could be alleviated *via* psychological intervention (7, 8). Breast cancer-related fatigue is also a major issue influencing quality of life, and some rehabilitation protocols like aerobic exercise could help (9). Several studies were focused on prevalence of mental illness during surgical intervention or medical treatment after diagnosing breast cancer since many cases of breast cancer are associated with physical disability and a poor quality of life after their breast cancer diagnosis with symptoms of fatigue, depression, and anxiety (10). However, herein, we focused on the association of insomnia, depressive disorders, or mood disorders with subsequent breast cancer in women.

Insomnia, one of the most common sleep disorders, affects approximately 6%–30% of the overall population (11–13). Emerging evidence suggests that insomnia is independently associated with psychiatric diseases (14), impaired health-related quality of life (15), and increased risks of hormone-related diseases (16) such as cancers (17).

The mood disorders are currently confined to disorders in which the mood is depressed or elevated. Mood disorders have once been interchangeably viewed as “affective disorder”, a term which is still used frequently. Some studies have revealed that depressive disorders are related to substantial mortality, some comorbidities, and disabilities (18, 19).

Globally, around 10.7% of disability can be attributed to unipolar major depression. According to Joyce (20), unipolar major depression accounts for nearly 20% of disease burden in women aged 15 to 44 years old in developed countries. Therefore, we also investigated the association of some variants like insured amount, level of urbanization, and residential location in representatives of socioeconomic status, with breast cancer.

## Methods

### Data Sources/Measurement

The Taiwan National Health Insurance Research Database (NHIRD) was established in 1995. The compulsory National Health Insurance program covered more than 99% of Taiwan residents, and their original claim data were stored in NHIRD. We utilized and analyzed the Longitudinal Health Insurance Data (LHID), which contains data of one million random selected insureds. The medical data included outpatient and inpatient records, the medication used, and treatment received. The identification number was encoded for protecting privacy problems. The diagnostic codes were recorded based on the International Classification of Disease, Ninth Revision, Clinical Modification (ICD-9-CM). This study was approved by the ethical review board of the China Medical University Hospital [CMUH104-REC2-115(AR-4)].

### Study Cohort

**Figure 1:**
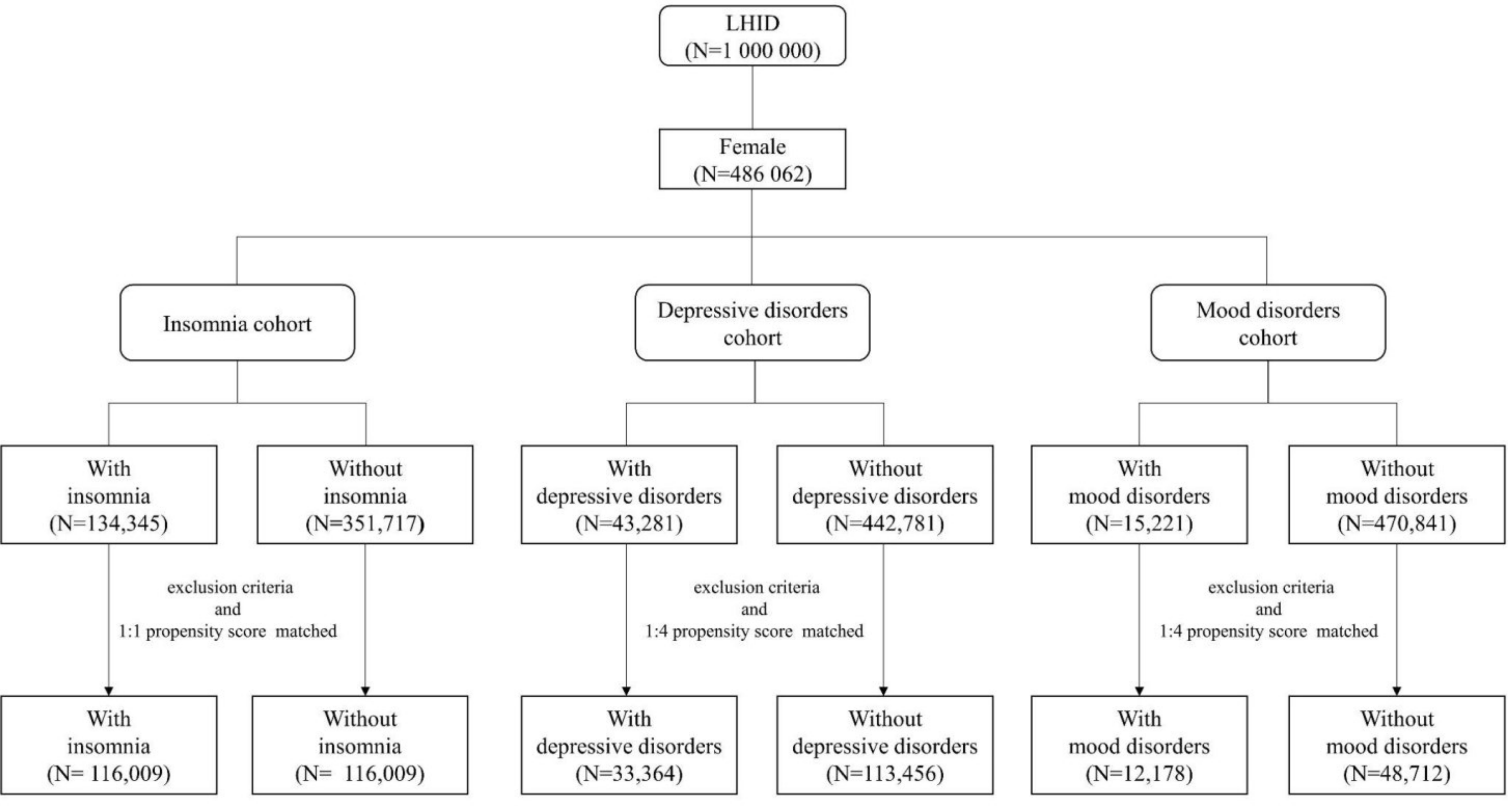
The flowchart of the three cohorts in this study: insomnia cohort, depressive disorder cohort, and mood disorder cohort.

In this study, we conducted three cohort studies which are shown in **Figure 1**. The first cohort was an insomnia cohort. Patients who were newly diagnosed with insomnia (ICD-9-CM codes 307.41, 307.42, 307.49, 780.50, 780.52, 780.55, 780.56, and 780.59) were the cases in this cohort. Those without insomnia were the controls. For the second cohort, the case cohort consisted of the patients with depressive disorders (ICD-9-CM codes 296.2, 296.3, 298.0, 300.4, 311, and V79.0) and the control cohort were patients never diagnosed with depressive disorders. The last cohort recruited patients with mood disorders (ICD-9-CM codes 296.0–296.7) as the case cohort and patients without mood disorders as the control cohort. The first diagnosis was the index date for case cohort and a random date between 2000 and 2012 was assigned to controls as the index date. The study period for all cohorts was 2000 to 2013. We excluded patients who were male, aged below 20 and diagnosed with breast cancer before the index date. Control patients were matched to case patients according to age, insured amount, urbanization level, residential location, and index year in a 1:1 ratio for the insomnia cohort and in 1:4 ratio for depressive disorder and mood disorder cohorts.

### Primary Outcome

Breast cancer (ICD-9-CM code 174) was the main outcome in this study. Patients who received the Major Illness or Injury Certificate of breast cancer were defined as the outcome. We followed up the participants from the index date to the development of breast cancer; withdraw from the NHI program or the end of the study, Dec. 31, 2013.

### Variables

Demographic variables included age, insured amount, urbanization, and residential location. Age was divided into four groups: 20–30 years old; 31–40 years old; 41–50 years old; and >50 years old. The related comorbidities included chronic obstructive pulmonary disease (COPD) (ICD-9-CM codes 491, 492, and 496), hypertension (HTN) (ICD-9-CM codes 401–405), diabetes mellitus (DM) (ICD-9-CM code 250), chronic kidney disease (CKD) (ICD-9-CM code 585), and hyperlipidemia (ICD-9-CM code 272). We also considered the use of sleeping pills potential confounders.

### Statistical Methods

The difference of the categorical variables and continuous variables in case cohort and the control cohort were expressed by standard mean difference (SMD). SMD of less than 0.1 means the difference can be neglected. The hazard ratio (HR) and 95% confidence interval (CI) were calculated by the Cox proportional model. The Kaplan-Meier method was applied to obtain the cumulative incidence curves and tested by the Log-rank test. All statistical analysis was performed by software SAS (version 9.4 for Windows; SAS Institute, Inc., Cary, NC, USA). A *p*-value of less than 0.05 was the statistically significant level.

## Results

### Patient Characteristics

#### With and Without Insomnia

Baseline characteristics of the 116,009 women with noninsomnia and 116,009 women with insomnia in this cohort (mean [SD] age: 47.8 [16.4] in noninsomnia group and 47.6 [15.7] in insomnia group) are provided in **Table 1**. The insured amount within 10,000–20,000 was predominant in noninsomnia [36%] and insomnia [38%]. Patients were mainly in highly urbanized areas in the noninsomnia (69,759 [60%]) and insomnia (70,372 [61%]) groups. The majority of patients (noninsomnia (52,296 [45%]) and insomnia (51,792 [45%])) resided in the northern area of Taiwan. Compared with patients with noninsomnia, those with insomnia had more comorbidities including COPD (18,879 [16%] vs. 28,271 [24%]), HTN (24,046 [21%] vs. 30,609 [26%]), CKD (7,558 [7%] vs. 11,403 [10%]), and hyperlipidemia (16,296 [14%] vs. 22,425 [19%]) (SMD >0.1 for all). Compared with patients with noninsomnia, those with insomnia had more sleeping pills (21,999 [19%] vs. 34,612 [30%]) (SMD >0.1).

**Table 1:** The baseline characteristics of patients with and without insomnia.

Variable	Noninsomnia		Insomnia		SMD
	*N* = 116,009		*N* = 116,009		
	*n*	%	*n*	%	
Age (year)					
20–30	17,695	15%	17,381	15%	0.008
30–40	23,150	20%	22,677	20%	0.010
40–50	27,524	24%	27,708	24%	0.004
>50	47,640	41%	48,243	42%	0.001
Mean (SD)	47.8	(16.4)	47.6	(15.7)	0.011
Insured amount (TWD)					
≤10,000	40,126	35%	39,306	34%	0.015
10,000–20,000	42,241	36%	43,756	38%	0.027
>20,000	33,642	29%	32,947	28%	0.013
Urbanization					
Low	9,136	8%	8,827	8%	0.010
Medium	37,114	32%	36,810	32%	0.006
High	69,759	60%	70,372	61%	0.011
Residential location					
Northern	52,296	45%	51,792	45%	0.009
Central	28,835	25%	31,369	27%	0.050
Southern	13,846	12%	12,882	11%	0.026
Eastern	20,790	18%	19,792	17%	0.023
Others	242	0.21%	174	0.15%	0.014
Comorbidities					
COPD	18,879	16%	28,271	24%	0.202
HTN	24,046	21%	30,609	26%	0.134
DM	11,910	10%	14,486	12%	0.070
CKD	7,558	7%	11,403	10%	0.121
Hyperlipidemia	16,296	14%	22,425	19%	0.142
Medication					
Sleep pills	21,999	19%	34,612	30%	0.255

COPD, chronic obstruction pulmonary disease; HTN, hypertension; DM, diabetes mellitus; CKD, chronic kidney disease; SMD, standard mean difference (less than 0.1 means no difference).

#### With and Without Depressive Disorders

**Table 2** presents the baseline characteristics of 33,364 women with depressive disorders and 133,456 women without depressive disorders (mean [SD] age: 48.1 [16.9] in nondepressive disorder group and 48.2 [16.8] in depressive disorder group). Most patients were in highly urbanized areas (83,346 [62%]) for the control group and 20,835 [62%] for depressive disorders). Patients with depressive disorders had more comorbidities including COPD (25,314 [19%] vs. 9,200 [28%]), HTN (31,182 [23%] vs. 10,350 [31%]), DM (15,243 [11%] vs. 5,280 [16%]), CKD (10,314 [8%] vs. 4,094 [12%]), and hyperlipidemia (21,846 [16%] vs. 7,866 [24%]) (SMD >0.1) than those without depressive disorders.

**Table 2:** The baseline characteristics of patients with and without depressive disorder.

Variable	Nondepressive disorders		Depressive disorders		SMD
	*N* = 133,456		*N* = 33,364		
	*n*	%	*n*	%	
Age (year)					
20–30	21,988	16%	5,484	16%	0.001
30–40	25,693	19%	6,390	19%	0.003
40–50	28,570	21%	7,057	21%	0.006
>50	57,205	43%	14,433	43%	0.008
Mean (SD)	48.1	(16.9)	48.2	(16.8)	0.005
Insured amount (TWD)					
≤10,000	48,189	36%	12,013	36%	0.002
10,000–20,000	49,638	37%	12,494	37%	0.005
>20,000	35,629	27%	8,857	27%	0.003
Urbanization					
Low	9,566	7%	2,577	8%	0.021
Medium	40,544	30%	9,952	30%	0.012
High	83,346	62%	20,835	62%	<0.001
Residential location					
Northern	61,277	46%	15,248	46%	0.004
Central	30,909	23%	7,700	23%	0.002
Southern	15,474	12%	3,886	12%	0.002
Eastern	25,599	19%	6,479	19%	0.006
Others	197	0.15%	51	0.15%	0.001
Comorbidities					
COPD	25,314	19%	9,200	28%	0.205
HTN	31,182	23%	10,350	31%	0.173
DM	15,243	11%	5,280	16%	0.129
CKD	10,314	8%	4,094	12%	0.152
Hyperlipidemia	21,846	16%	7,866	24%	0.181
Medication					
Sleep pills	32,305	24%	8,250	25%	0.012

COPD, chronic obstruction pulmonary disease; HTN, hypertension; DM, diabetes mellitus; CKD, chronic kidney disease; SMD, standard mean difference (less than 0.1 means no difference).

#### With and Without Mood Disorders

As shown in **Table 3**, 12,178 women with mood disorders and 48,712 patients without mood disorders (mean [SD] age: 46.6 [16.5%] in nonmood disorder group and 46.6 [16.4%] in depressive disorder group) are included. A higher proportion of comorbidities including COPD (9,165 [19%] vs. 3,480 [29%]), HTN (10,710 [22%] vs. 3,645 [30%]), DM (5,443 [11%] vs. 1,971 [16%]), CKD (3,661 [8%] vs. 1,519 [12%]), and hyperlipidemia (7,874 [16%] vs. 2,842 [23%]) were found in patient with mood disorders.

**Table 3:** The baseline characteristics of patients with and without mood disorders.

Variable	Nonmood disorders		Mood disorders		SMD
	*N* = 48,712		*N* = 12,178		
	*n*	%	*n*	%	
Age (year)					
20–30	8,954	18%	2,238	18%	<0.001
30–40	10,295	21%	2,565	21%	0.002
40–50	10,320	21%	2,576	21%	0.001
>50	19,143	39%	4,799	39%	0.002
Mean (SD)	46.6	(16.5)	46.6	(16.4)	0.002
Insured amount (TWD)					
≤10,000	18,749	39%	4,690	39%	<0.001
10,000–20,000	18,555	38%	4,646	38%	0.001
>20,000	11,408	23%	2,842	23%	0.002
Urbanization					
Low	3,329	7%	871	7%	0.012
Medium	14,071	29%	3,510	29%	0.001
High	31,312	64%	7,797	64%	0.005
Residential location					
Northern	23,031	47%	5,756	47%	<0.001
Central	10,815	22%	2,700	22%	0.001
Southern	4,901	10%	1,225	10%	<0.001
Eastern	9,933	20%	2,484	20%	<0.001
Others	32	0.07%	13	0.11%	0.014
Comorbidities					
COPD	9,165	19%	3,480	29%	0.231
HTN	10,710	22%	3,645	30%	0.182
DM	5,443	11%	1,971	16%	0.146
CKD	3,661	8%	1,519	12%	0.166
Hyperlipidemia	7,874	16%	2,842	23%	0.181
Medication					
Sleep pills	11,665	24%	2,367	19%	0.110

COPD, chronic obstruction pulmonary disease; HTN, hypertension; DM, diabetes mellitus; CKD, chronic kidney disease; SMD, standard mean difference (less than 0.1 means no difference).

### The Incidence Rate and Hazard Ratios of Breast Cancer

#### With and Without Insomnia

**Figure 2:**
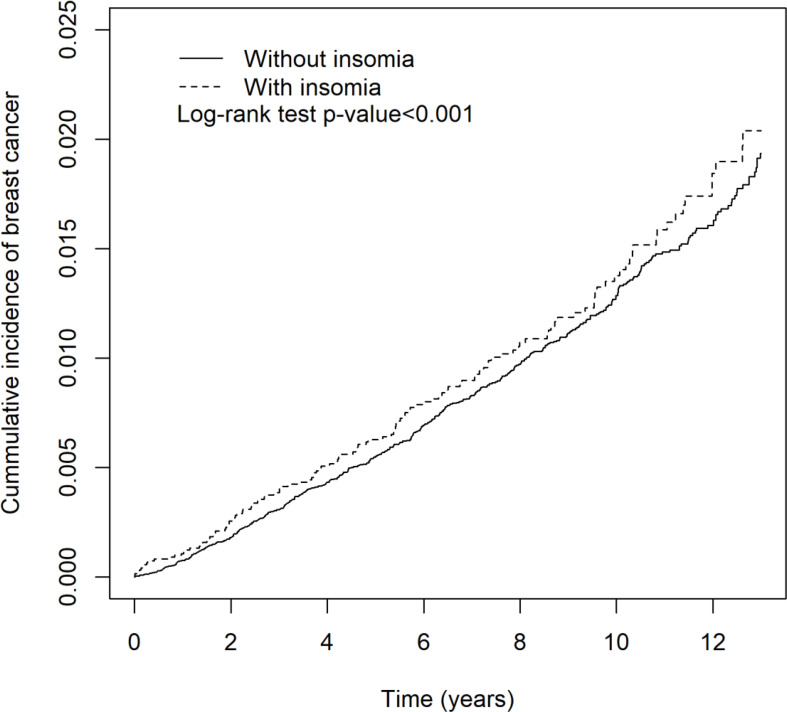
The cumulative incidence of breast cancer in patients with and without insomnia.

**Table 4** shows that women with insomnia had increased breast cancer (aHR = 1.16 (95% CI = 1.07, 1.27), *p* < 0.001). The cumulative incidence of breast cancer in patients with and without insomnia is shown in **Figure 2**. Patients with sleep medication were associated with a significantly increased incidence rate of breast cancer (aHR = 1.23 (95% CI = 1.13, 1.35), *p* < 0.001). Annual insured amount >20,000 (TWD), high urbanization area, and hyperlipidemia were associated with increased hazard of breast cancer (aHR = 1.13 (95% CI = 1.01, 1.27), *p* = 0.04; aHR = 1.41 (95% CI = 1.17, 1.71), *p* < 0.001; aHR = 1.14 (95% CI = 1.02, 1.29), *p* = 0.02, respectively).

**Table 4:** The incidence rates and hazard ratios of breast cancer in insomnia cohort.

Variable	Breast cancer			cHR (95% CI)	aHR^a^ (95% CI)
	n	PY	IR		
Insomnia					
No	1,023	874,576	1.17	1.00 (–)	1.00 (–)
Yes	1,257	892,053	1.41	1.21 (1.12, 1.32)^***^	1.16 (1.07, 1.27)^***^
Age (year)					
20–30	47	257,668	0.18	1.00 (–)	1.00 (–)
30–40	339	354,040	0.96	5.16 (3.80, 7.00)^***^	4.80 (3.52, 6.54)^***^
40–50	801	446,414	1.79	9.57 (7.13, 12.8)^***^	8.64 (6.39, 11.7)^***^
>50	1,093	708,507	1.54	8.28 (6.18, 11.1)^***^	7.55 (5.59, 10.2)^***^
Insured amount (TWD)					
≤10,000	489	554,518	0.88	1.00 (–)	1.00 (–)
10,000–20,000	970	678,151	1.43	1.58 (1.42, 1.76)^***^	1.10 (0.99, 1.23)
>20,000	821	533,960	1.54	1.68 (1.50, 1.88)^***^	1.13 (1.01, 1.27)^*^
Urbanization					
Low	130	139,811	0.93	1.00 (–)	1.00 (–)
Medium	678	565,687	1.20	1.31 (1.09, 1.59)^**^	1.27 (1.05, 1.54)^*^
High	1,472	1,061,131	1.39	1.52 (1.27, 1.82)^***^	1.41 (1.17, 1.71)^***^
Residential location					
Northern	1,122	790,556	1.42	1.00 (–)	1.00 (–)
Central	554	463,092	1.20	0.84 (0.76, 0.93)^**^	0.95 (0.85, 1.06)
Southern	254	200,251	1.27	0.89 (0.78, 1.02)	0.96 (0.84, 1.11)
Eastern	348	309,410	1.12	0.79 (0.70, 0.89)^***^	0.86 (0.76, 0.97)^*^
Others	2	3,320	0.60	0.42 (0.10, 1.68)	0.57 (0.14, 2.33)
Comorbidities					
COPD					
No	1,819	1,432,028	1.27	1.00 (–)	
Yes	461	334,601	1.38	1.10 (1.00, 1.22)	
HTN					
No	1,676	1,373,140	1.22	1.00 (–)	1.00 (–)
Yes	604	393,490	1.53	1.27 (1.16, 1.39)^***^	0.96 (0.86, 1.07)
DM					
No	1,991	1,581,543	1.26	1.00 (–)	1.00 (–)
Yes	289	185,087	1.56	1.27 (1.12, 1.44)^***^	1.00 (0.88, 1.15)
CKD					
No	2,096	1,626,558	1.29	1.00 (–)	
Yes	184	140,072	1.31	1.02 (0.88, 1.19)	
Hyperlipidemia					
No	1,826	1,493,915	1.22	1.00 (–)	1.00 (–)
Yes	454	272,715	1.66	1.40 (1.27, 1.56)^***^	1.14 (1.02, 1.29)^*^
Medication					
Sleep pills					
No	1,446	1,243,629	1.16	1.00 (–)	1.00 (–)
Yes	834	523,001	1.59	1.28 (1.18, 1.4)^***^	1.23 (1.13, 1.35)^***^

^*^p-value <0.05; ^**^p-value <0.01; ^***^p-value <0.001.

PY, person-years; IR, incidence rate (per 1,000 person-years); cHR, crude hazard ratio; aHR, adjusted hazard ratio; COPD, chronic obstruction pulmonary disease; HTN, hypertension; DM, diabetes mellitus; CKD, chronic kidney disease.

a Adjusted by age, insured amount, HTN, DM, hyperlipidemia, and sleep pills.

#### With and Without Depressive Disorders/Mood Disorders

**Figure 3:**
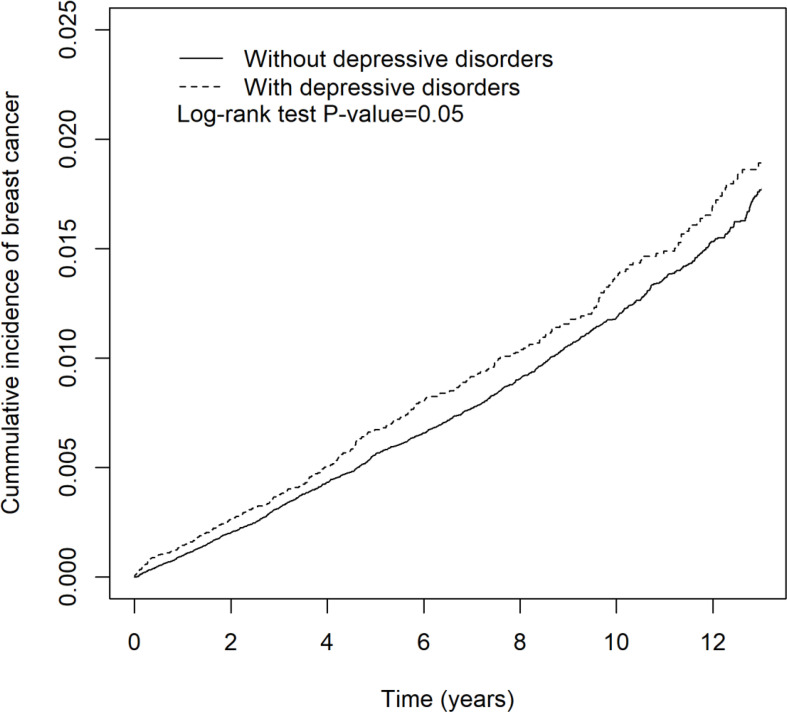
The cumulative incidence of breast cancer in patients with and without depressive disorders.

**Figure 4:**
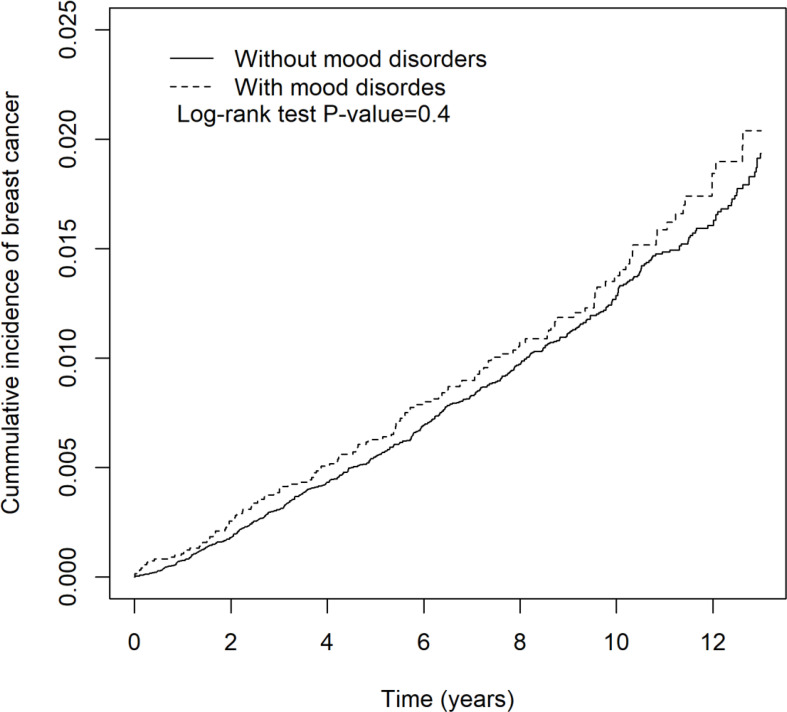
The cumulative incidence of breast cancer in patients with and without mood disorders.

The depressive disorder- or mood disorder-related incidence rates and hazard ratio of breast cancer are shown in **Tables 5** and **6**, respectively. In **Table 5**, there was a positive correlation between depressive disorders and increased incidence rate of breast cancer, but it was not statistically significant (aHR = 1.11 (95% CI = 0.99, 1.25), *p* = 0.08). Patients with sleep medication were associated with a significantly increased incidence rate of breast cancer (aHR = 1.39 (95% CI = 1.26, 1.54), *p* < 0.001). High urbanization area and hyperlipidemia were associated with increased hazard of breast cancer (aHR = 1.37 (95% CI = 1.10, 1.71), *p* < 0.01; aHR = 1.32 (95% CI = 1.15, 1.51), *p* < 0.001, respectively). In **Table 6**, the patients with mood disorders were not associated with increased hazard of breast cancer (aHR = 1.11 (95% CI = 0.91, 1.34), *p* = 0.31). Patients with sleep medication were associated with a significant increased incidence rate of breast cancer (aHR = 1.53 (95% CI = 1.30, 1.80), *p* < 0.001). Annual insured amount >20,000 (TWD) and hyperlipidemia were associated with increased hazard of breast cancer (aHR = 1.24 (95% CI = 1.00, 1.54), *p* < 0.05; aHR = 1.33 (95% CI = 1.06, 1.66), *p* < 0.001, respectively). Kaplan-Meier curves of breast cancer in the depressive disorder cohort and the mood disorder cohort are demonstrated in **Figures 3** and **4**, individually.

**Table 5:** The incidence rates and hazard ratios of breast cancer in depressive disorder cohort.

Variable	Breast cancer			cHR (95% CI)	aHR^a^ (95% CI)
	n	PY	IR		
Depressive disorders					
No	1,308	1,042,122	1.26	1.00 (–)	1.00 (–)
Yes	360	257,472	1.40	1.12 (1.00, 1.26)	1.11 (0.99, 1.25)
Age (year)					
20–30	39	225,057	0.17	1.00 (–)	1.00 (–)
30–40	262	265,806	0.99	5.76 (4.1, 8.09)^***^	5.36 (3.79, 7.58)^***^
40–50	537	293,100	1.83	10.8 (7.79, 15.04)^***^	9.71 (6.93, 13.6)^***^
>50	830	515,631	1.61	9.68 (6.99, 13.4)^***^	8.54 (6.10, 12.0)^***^
Insured amount (TWD)					
≤10,000	394	444,643	0.89	1.00 (–)	1.00 (–)
10,000–20,000	710	494,198	1.44	1.60 (1.42, 1.81)^***^	1.10 (0.97, 1.25)
>20,000	564	360,753	1.56	1.74 (1.53, 1.98)^***^	1.13 (0.99, 1.29)
Urbanization					
Low	93	94,619	0.98	1.00 (–)	1.00 (–)
Medium	439	389,455	1.13	1.14 (0.91, 1.43)	1.15 (0.92, 1.45)
High	1,136	815,520	1.39	1.42 (1.15, 1.75)^**^	1.37 (1.10, 1.71)^**^
Residential location					
Northern	843	598,870	1.41	1.00 (–)	1.00 (–)
Central	353	301,025	1.17	0.82 (0.72, 0.93)^**^	0.89 (0.78, 1.02)
Southern	161	150,151	1.07	0.76 (0.64,0.9)^**^	0.81 (0.68, 0.96)^*^
Eastern	311	247,579	1.26	0.89 (0.79, 1.02)	0.95 (0.83, 1.09)
Others	0	1,970	0.00	0.00 (0, Inf)	
Comorbidities					
COPD					
No	1,323	1,065,561	1.24	1.00 (–)	1.00 (–)
Yes	345	234,033	1.47	1.24 (1.1, 1.4)^***^	1.05 (0.92, 1.18)
HTN					
No	1,204	1,017,375	1.18	1.00 (–)	1.00 (–)
Yes	464	282,219	1.64	1.44 (1.29, 1.6)^***^	1.02 (0.90, 1.16)
DM					
No	1,466	1,164,932	1.26	1.00 (–)	1.00 (–)
Yes	202	134,662	1.50	1.24 (1.07, 1.44)^**^	0.86 (0.73, 1.01)
CKD					
No	1,511	1,199,194	1.26	1.00 (–)	1.00 (–)
Yes	157	100,400	1.56	1.27 (1.07, 1.49)^**^	1.01 (0.85, 1.19)
Hyperlipidemia					
No	1,307	1,102,930	1.19	1.00 (–)	1.00 (–)
Yes	361	196,664	1.84	1.63 (1.45, 1.83)^***^	1.32 (1.15, 1.51)^***^
Medication					
Sleep pills					
No	1,008	903,466	1.12	1.00 (–)	1.00 (–)
Yes	660	396,128	1.67	1.41 (1.28, 1.56)^***^	1.39 (1.26, 1.54)^***^

^*^p-value <0.05; ^**^p-value <0.01; ^***^p-value <0.001.

PY, person-years; IR, incidence rate (per 1,000 person-years); cHR, crude hazard ratio; aHR, adjusted hazard ratio; COPD, chronic obstruction pulmonary disease; HTN, hypertension; DM, diabetes mellitus; CKD, chronic kidney disease.

a Adjusted by age, insured amount, COPD, HTN, DM, CKD, hyperlipidemia, and sleep pills.

**Table 6:** The incidence rates and hazard ratios of breast cancer in mood disorder cohort.

Variable	Breast cancer			cHR (95% CI)	aHR^a^ (95% CI)
	n	PY	IR		
Mood disorders					
No	495	375,364	1.32	1.00 (–)	1.00 (–)
Yes	131	91,702	1.43	1.09 (0.90, 1.32)	1.11 (0.91,1.34)
Age (year)					
20–30	14	90,695	0.15	1.00 (–)	1.00 (–)
30–40	129	104,577	1.23	7.86 (4.53, 13.7)^***^	7.30 (4.16,12.8)^***^
40–50	189	103,438	1.83	11.7 (6.81, 20.2)^***^	10.3 (5.92,18.0)^***^
>50	294	168,356	1.75	11.5 (6.73, 19.7)^***^	9.72 (5.57, 16.9)^***^
Insured amount (TWD)					
≤10,000	153	171,782	0.89	1.00 (–)	1.00 (–)
10,000–20,000	261	179,997	1.45	1.61 (1.32, 1.96)^***^	1.08 (0.88, 1.32)
>20,000	212	115,288	1.84	2.01 (1.63, 2.47)^***^	1.24 (1.00, 1.54)^*^
Urbanization					
Low	41	33,127	1.24	1.00 (–)	
Medium	152	132,318	1.15	0.94 (0.66, 1.32)	
High	433	301,621	1.44	1.17 (0.85, 1.61)	
Residential location					
Northern	314	222,849	1.41	1.00 (–)	
Central	139	102,413	1.36	0.97 (0.79, 1.18)	
Southern	55	45,434	1.21	0.87 (0.65, 1.16)	
Eastern	116	95,951	1.21	0.86 (0.69, 1.06)	
Others	2	420	4.76	3.27 (0.81, 13.1)	
Comorbidities					
COPD					
No	489	382,740	1.28	1.00 (–)	1.00 (–)
Yes	137	84,326	1.62	1.33 (1.1, 1.61)^**^	1.11 (0.91, 1.35)
HTN					
No	463	370,835	1.25	1.00 (–)	1.00 (–)
Yes	163	96,231	1.69	1.41 (1.18, 1.69)^***^	0.90 (0.73, 1.12)
DM					
No	531	418,970	1.27	1.00 (–)	1.00 (–)
Yes	95	48,096	1.98	1.63 (1.31, 2.03)^***^	1.17 (0.91, 1.50)
CKD					
No	569	431,056	1.32	1.00 (–)	
Yes	57	36,010	1.58	1.22 (0.93, 1.60)	
Hyperlipidemia					
No	486	397,676	1.22	1.00 (–)	1.00 (–)
Yes	140	69,390	2.02	1.74 (1.44, 2.10)^***^	1.33 (1.06, 1.66)^*^
Medication					
Sleep pills					
No	379	331,365	1.14	1.00 (–)	1.00 (–)
Yes	247	135,701	1.82	1.50 (1.28, 1.77)^***^	1.53 (1.30, 1.80)^***^

^*^p-value <0.05; ^**^p-value <0.01; ^***^p-value <0.001.

PY, person-years; IR, incidence rate (per 1,000 person-years); cHR, crude hazard ratio; aHR, adjusted hazard ratio; COPD, chronic obstruction pulmonary disease; HTN, hypertension; DM, diabetes mellitus; CKD, chronic kidney disease.

a Adjusted by age, insured amount, COPD, HTN, DM, hyperlipidemia, and sleep pills.

### The Association of Insomnia and Breast Cancer in Different Stratification

Patients with insomnia along with annual insured amount of more than 20,000 (TWD) (aHR = 1.27 (95% CI = 1.10, 1.46), *p* < 0.001) and high urbanization (aHR = 1.23 (95% CI = 1.11, 1.37), *p* < 0.001) were associated with significant increased hazard of breast cancer relative to those without insomnia, see **Table 7**. Insomnia patients with residential location in the northern area also increased the risk of breast cancer by 1.22 times (95% CI = 1.08, 1.38; *p* = 0.001) compared with the control patients who lived in the northern area. Patients who had insomnia with sleep medication did not yield an increased hazard of breast cancer over those without insomnia.

**Table 7:** The association of insomnia and breast cancer in different stratifications.

Variable	Noninsomnia			Insomnia			cHR (95% CI)	aHR^a^ (95% CI)
	n	PY	IR	n	PY	IR		
Age (year)								
20–30	24	130,189	0.18	23	127,479	0.18	1.05 (0.59, 1.87)	1.12 (0.63, 2.01)
30–40	147	179,431	0.82	192	174,609	1.10	1.37 (1.10, 1.70)^**^	1.31 (1.05, 1.63)^*^
40–50	372	223,081	1.67	429	223,334	1.92	1.16 (1.01, 1.33)^*^	1.11 (0.84, 1.26)
>50	480	341,875	1.40	613	366,632	1.67	1.20 (1.06, 1.35)^**^	1.17 (1.04, 1.32)^*^
Insured amount (TWD)								
≤10,000	228	277,420	0.82	261	277,098	0.94	1.15 (0.96, 1.37)	1.10 (0.91, 1.31)
10,000–20,000	434	328,620	1.32	536	349,531	1.53	1.16 (1.02, 1.32)^*^	1.12 (0.98, 1,27)
>20,000	361	268,536	1.34	460	265,424	1.73	1.31 (1.14, 1.51)^***^	1.27 (1.10, 1.46)^***^
Urbanization								
Low	50	69,996	0.71	80	69,815	1.15	1.69 (1.18, 2.41)^**^	1.62 (1.12, 2.33)^**^
Medium	336	280,454	1.20	342	285,233	1.20	1.00 (0.86, 1.16)	0.96 (0.82, 1.12)
High	637	524,126	1.22	835	537,005	1.55	1.29 (1.16, 1.43)^***^	1.23 (1.11, 1.37)^***^
Residential location								
Northern	498	394,530	1.26	624	396,026	1.58	1.26 (1.12, 1.42)^***^	1.22 (1.08, 1.38)^**^
Central	233	216,425	1.08	321	246,668	1.30	1.20 (1.01, 1.42)^*^	1.14 (0.96, 1.35)
Southern	117	103,864	1.13	137	96,387	1.42	1.30 (1.01, 1.66)^*^	1.23 (0.95, 1.58)
Eastern	173	157,823	1.10	175	151,587	1.15	1.07 (0.86, 1.32)	1.00 (0.80, 1.23)
Others	2	1,935	1.03	0	1,386	0.00		
Comorbidities								
COPD								
No	864	749,752	1.15	955	682,276	1.40	1.22 (1.12, 1.34)^***^	1.17 (1.07, 1.29)^***^
Yes	159	124,824	1.27	302	209,777	1.44	1.12 (0.92, 1.35)	1.12 (0.92, 1.36)
HTN								
No	794	713,200	1.11	882	659,939	1.34	1.21 (1.10, 1.34)^***^	1.18 (1.07, 1.30)^***^
Yes	229	161,376	1.42	375	232,114	1.62	1.13 (0.96, 1.34)	1.11 (0.94, 1.31)
DM								
No	917	796,971	1.15	1,074	784,571	1.37	1.20 (1.10, 1.31)^***^	1.15 (1.05, 1.26)^**^
Yes	106	77,605	1.37	183	107,482	1.70	1.21 (0.96, 1.54)	1.20 (0.95, 1.53)
CKD								
No	963	823,377	1.17	1,133	803,181	1.41	1.22 (1.12, 1.33)^***^	1.17 (1.07, 1.28)^***^
Yes	60	51,200	1.17	124	88,872	1.40	1.19 (0.87, 1.62)	1.16 (0.85, 1.59)
Hyperlipidemia								
No	860	766,579	1.12	966	727,336	1.33	1.20 (1.09, 1.31)^***^	1.16 (1.06, 1.28)^**^
Yes	163	107,997	1.51	291	164,717	1.77	1.16 (0.95, 1.40)	1.16 (0.96, 1.41)
Medication								
Sleep pills								
No	682	666,869	1.02	764	576,760	1.32	1.31 (1.18, 1.45)^***^	1.27 (1.15, 1.42)^***^
Yes	341	207,707	1.64	493	315,293	1.56	0.97 (0.85, 1.12)	0.97 (0.84, 1.11)

^*^p-value <0.05; ^**^p-value <0.01; ^***^p-value <0.001.

PY, person-years; IR, incidence rate (per 1,000 person-years); cHR, crude hazard ratio; aHR, adjusted hazard ratio; COPD, chronic obstruction pulmonary disease; HTN, hypertension; DM, diabetes mellitus; CKD, chronic kidney disease.

a Adjusted by age, insured amount, hypertension, diabetes, hyperlipidemia, and sleep pills.

### The Effect of Sleeping Pills on Breast Cancer in Insomnia Cohort

The effect of sleeping pills on breast cancer is shown in **Table 8**. Either noninsomnia with sleeping pills (aHR = 1.46 (95% CI = 1.28, 1.67), *p* < 0.001), insomnia without sleeping pills (aHR = 1.28 (95% CI = 1.16, 1.42), *p* < 0.001), or insomnia with sleeping pills (aHR = 1.43 (95% CI = 1.27, 1.61), *p* < 0.001) was associated with significant increased hazard of breast cancer. However, insomnia combined with sleeping pills did not yield more hazard ratio than either insomnia or sleeping pills alone.

**Table 8:** The effect of sleep pills on breast cancer in insomnia cohort.

Insomnia	Sleep pills	*N*	*n*	cHR (95% CI)	aHR^a^ (95% CI)
No	No	94,010	682	1.00 (–)	1.00 (–)
No	Yes	21,999	341	1.47 (1.29, 1.68)^***^	1.46 (1.28, 1.67)^***^
Yes	No	81,397	764	1.30 (1.18, 1.45)^***^	1.28 (1.16, 1.42)^***^
Yes	Yes	34,612	493	1.45 (1.29, 1.63)^***^	1.43 (1.27, 1.61)^***^

^***^p-value <0.001.

N, number of people; n, number of breast cancer; cHR, crude hazard ratio; aHR, adjusted hazard ratio. ^a^Adjusted by age, insured amount, hypertension, diabetes, hyperlipidemia, and sleep pills.

## Discussion

To our knowledge, the present analysis, which pooled data from the Taiwan National Health Insurance Research Database, is the largest study to evaluate breast cancer and insomnia, depressive disorders, and mood disorders. In this cohort study, women (>20 years old) with insomnia had 16% higher rates of breast cancer. We also found that sleep medication, high insured amount, highly urbanized areas, and hyperlipidemia were associated with significantly increased incidence rate of breast cancer. There was a positive correlation between depressive disorders and increased incidence rate of breast cancer, but it was not statistically significant. Mood disorders were not associated with increased hazard of breast cancer. In summary, the risk factors of breast cancer in our study were insomnia, sleep medication, high urbanization, high insured amount, and hyperlipidemia.

Several studies concerning insomnia were mainly focused on the relationship of patients with preexisting breast cancer, but insomnia as a risk factor for breast cancer among primarily cancer-free women were rarely investigated (17, 21). Shift work involving circadian disruption was classified as being a probable carcinogen to humans according to the World Health Organization’s International Agency for Research on Cancer in 2007 (22). The likely underlying mechanisms could be disturbed sleep, exposure to light at night, and other lifestyle factors (23–25). A positive correlation between circadian disruption and breast cancer (HR = 1.14 (95% CI = 1.08, 1.21)) was proposed in a meta-analysis of 28 studies. Whereas, short sleep duration (<7 h/night) and dose-response association with sleep deficiency were not conclusive (26). Neither chronotype nor individual insomnia symptoms were strongly associated with breast cancer in evidence (27, 28). The relationship between insomnia and breast cancer could be understood *via* metabolic dysfunction as glucose homeostasis imbalance. Those with elevated blood glucose were related to decreased survival independent of comorbid diabetes mellitus (type 2) and body mass index (29, 30). According to Borniger et al., in a single nonmetastatic model of breast cancer, the underlying mechanism mediating cancer-associated metabolic changes and sleep disruption may be aberrant activity of wake-stabilizing hypocretin/orexin (HO) neuron (31).

Sleep medication, also called hypnotics, are mainly divided into benzodiazepines (BZDs) and nonbenzodiazepines (non-BZDs), and other miscellaneous types like gamma-aminobutyric acid agonist, melatonin receptor agonists, antihistamines, sedating antidepressant, and eugeroic drugs are not commonly administered (32). Both BZDs and non-BZDs (zolpidem, most widely used) were found with increased risk of cancers including breast cancer in two Taiwanese population-based cohort studies (33, 34). According to Iqbal et al. (34), it was provided based on epidemiological and bioinformatics analysis approaches that diazepam and zolpidem, but not alprazolam, might be associated with breast cancer risk. The underlying mechanisms between sleep medication and cancer remain obscure. Overexpression of peripheral benzodiazepine receptors (PBR) due to BZDs has been implicated in breast cancer (35), and cell proliferation was found in some breast cancer cell lines after administration of PBR agonist (36). *In vitro* studies, non-BZDs as zopiclone, zaleplon, and ramelteon were found clastogenic, which can lead to carcinogenesis *via* the process of inducing disruption or breakages of chromosomes (37).

Depressive disorders, featured by sadness or irritability, are prevalent chronic diseases with psycho-physiological symptoms (38). In breast cancer patients with comorbid anxiety and depression, a tendency of diagnosis delay beyond 90 days from symptom identification was reported. Furthermore, treatment delay of longer than 60 days from diagnosis establishment was remarkable in those with severe mental illness (39). According to Chen et al. (40), curative surgery of breast cancer was associated with increased risk of subsequent depressive disorders, and those who developed depressive disorders had higher incidence rate of tumor recurrence and mortality when followed up. There are some probable underlying mechanisms suggesting depressive disorders being carcinogenic, such as impairing immune function, giving rise to aberrancy of the hypothalamic-pituitary-adrenal axis and inhibiting DNA repair (41–43). Nevertheless, in a meta-analysis of cohort study in 2007, based on seven heterogeneous studies, showed there was no significant association between depression and subsequent breast cancer risk (44). Another recent meta-analysis of cohort study derived from 11 cohort studies in 2015 showed epidemiological evidence was insufficient to support a positive association between depression and breast cancer (45). In our study, we found a positive correlation between depressive disorders and increased incidence rate of breast cancer, but it was not statistically significant.

Mood disorders are great disease entities that include depressive disorders and bipolar disorders. Mood disorders in the *Diagnostic and Statistical Manual of Mental Disorders, fourth edition* (DSM-IV) have been replaced with multiple specifiers describing depressive disorders and bipolar disorders in the *Diagnostic and Statistical Manual of Mental Disorders, fifth edition* (DSM-V) (46). Also, DSM-IV entity of mood disorder NOS has been replaced with unspecified bipolar disorder and unspecified depressive disorder in DSM-V. To date, there were no previous studies aimed at the association between mood disorder and cancers. Since no remarkable association between bipolar disorders and breast cancer was noted in a nationwide cohort study (39), the association between depressive disorders and breast cancer was weak as mentioned above. It was hypothesized that mood disorders should be less coherent to breast cancer in that mood disorders are mainly partitioned into bipolar disorders and depressive disorders, and such inference was verified in our study, that is, mood disorders were not associated with increased hazard of breast cancer.

In high urbanization areas, in this study, the incidence rate of breast cancer in women was higher than in low urbanization areas. Also, a high insured amount was associated with increased breast cancer incidence. In a cohort study, high socioeconomic status and high income were found to have increased the incidence rate of breast cancer in the USA; however, income was not significantly correlated with mortality (47). In general, breast cancer has been regarded as a disease of affluence in risk factors such as delayed childbirth (48), less breastfeeding (49), and hormone supplements (50), which are more visible in affluent women. In one way, women in high socioeconomic areas have more accessibility to breast cancer screening as mammograms that detect more hidden cancer cases are highly available.

Hyperlipidemia is a status of chronic inflammation, which is a possible etiology contributing to breast cancer progression through activation of TLR signaling by oxidized LDL (51). In a hyperlipidemic mouse model, cholesterol and its metabolites have been verified to increase breast cell proliferation (52). In an observational study of 314 cases in China, elevated serum lipoprotein was associated with breast cancer (53). It is suggested that exercise could reduce the risk of hyperlipidemia-associated breast cancer *via* improving chronic inflammation, lowering blood lipid level, and exerting specific anticancer effects (54). In this study, we found that hyperlipidemia was an independent risk factor for breast cancer.

## Study Limitation

As this is a secondary analysis of observational data across the study period, we can only present the associations among breast cancer in Taiwanese women with insomnia, depressive disorders, mood disorders, sleep medication, insured amount, and hyperlipidemia. Also, we can only deduce cause and effect by showing that these associations are likely to be cohesive with a causal relationship between increased incidence of breast cancer and insomnia, sleep medication, high insured amount, highly urbanized areas, or hyperlipidemia. The results from this study, therefore, should be considered exploratory but preplanned and still need to be confirmed in subsequent clinical trials in the future. In this study, breast cancer, insomnia, depressive disorders, and mood disorders were based on ICD-9 codes which are replaced by ICD-10 codes currently. Because we focus on hypnotics in a general view instead of comparison of specific drugs, we could not differentiate the coefficient effect of different drugs. Routine mental illness screening is not popular in the Taiwan healthcare system. Besides, mental illnesses typically manifest years before people seek treatment (55); hence, the actual number of mental illness diagnosed before breast cancer could be underestimated, giving rise to inadequate interpretation of results.

## Conclusions

In this study, women with insomnia had increased risk of breast cancer, especially those in high urbanization or with high insured amounts. Sleep medication (BZD or non-BZD) and hyperlipidemia were independently associated with a higher hazard ratio of breast cancer. Insomnia combined with sleep medication did not yield more hazard as a synergic effect. Administration of sleeping pills should be less encouraged in women, if still needed, and they should be referred to a breast cancer department for regular screening, as are those diagnosed with insomnia. Mood disorders appeared to be not associated with subsequent breast cancer. However, depressive disorders, the subgroups of mood disorders, could possibly increase the incidence rate of breast cancer though not statistically significant. These findings emphasize the importance of early screening of breast cancer for patients with possible risk factors. Future studies evaluating differences in breast tumor cell type, differentiation, and genotype may help target interventions.

## Data Availability Statement

The raw data supporting the conclusions of this article will be made available by the authors, without undue reservation.

## Ethics Statement

The studies involving human participants were reviewed and approved by the China Medical University Hospital [CMUH104-REC2-115(AR-4)]. Written informed consent for participation was not required for this study in accordance with the national legislation and the institutional requirements.

## Author Contributions

Design: M-HY and JW. Acquisition of data: H-TY. Statistical analysis and interpretation: H-TY and H-PL. Manuscript writing: H-PL, JW, H-TY, and M-HY. All authors contributed to the article and approved the submitted version.

## Conflict of Interest

The authors declare that the research was conducted in the absence of any commercial or financial relationships that could be construed as a potential conflict of interest.

## Publisher’s Note

All claims expressed in this article are solely those of the authors and do not necessarily represent those of their affiliated organizations, or those of the publisher, the editors and the reviewers. Any product that may be evaluated in this article, or claim that may be made by its manufacturer, is not guaranteed or endorsed by the publisher.
